# O.5.1-1 Physical environment perceptions in rural and urban areas and their influence on adolescents’ walking and non-motorized vehicle use

**DOI:** 10.1093/eurpub/ckad133.231

**Published:** 2023-09-11

**Authors:** Leon Klos, Janis Fiedler, Carina Nigg, Alexander Burchartz, Thomas Hinz, Hagen Wäsche, Claudia Niessner, Alexander Woll

**Affiliations:** Institute of Sports and Sports Science, Karlsruhe Institute of Technology, Germany; Institute of Sports and Sports Science, Karlsruhe Institute of Technology, Germany; Institute of Sports and Sports Science, Karlsruhe Institute of Technology, Germany; Institute of Sports Science, University of Bern, Switzerland; Institute of Sports and Sports Science, Karlsruhe Institute of Technology, Germany; Department of History and Sociology, University of Konstanz, Germany; leon.klos@kit.edu; Institute of Sports and Sports Science, Karlsruhe Institute of Technology, Germany; Institute of Sports and Sports Science, Karlsruhe Institute of Technology, Germany; Institute of Sports and Sports Science, Karlsruhe Institute of Technology, Germany

## Abstract

**Purpose:**

Environment perceptions influence adolescents’ active transport. However, most studies focus on urban areas only and do not differentiate between walking and the use of non-motorized vehicles (NMV; e. g. cycling, skateboarding). Therefore, we aim to describe differences in perceived environment attributes across urbanicity levels and assess the relationship between perceived environment and walking as well as NMV in adolescents stratified across urban and rural areas.

**Methods:**

We used cross-sectional data including age, gender, socioeconomic status, environment perceptions, duration of walking, and NMV use of 3,976 adolescents (14.5±2.0 years, 51% female) from the nationwide Motorik-Modul Study Wave 2 (2014-2017) and Wave 3 pre-Covid (2018-2020) in Germany. Separate cumulative link mixed models were calculated for rural areas, small towns, medium-sized towns, and cities to analyze relationships between environment perceptions and walking as well as NMV use.

**Results:**

Adolescents in urban areas report higher presence of recreation facilities, walking and cycling infrastructure, cars, and children playing outside as well as better access to shops and bus stops compared to adolescents in rural areas. The latter perceive their neighborhood to be safer from crime and more pleasant for walking and cycling compared to urban areas. The relationships between environment perceptions and walking differ between urbanicity levels. For example, public sports facilities (OR = 1.29), cycling paths (OR = 1.30), and car presence are associated with walking in rural areas, whereas having access to shops (OR = 1.26) is associated with walking in cities. Rural-urban differences are also found for NMV use. For example, access to shops (OR = 1.16), cycling paths (OR = 1.29), and having a pleasant neighborhood for walking and cycling (OR = 1.29) are associated with NMV use in rural areas, whereas in cities, car presence (OR = 0.80) relates to NVT use. Public sports facilities (OR = 1.23-1.33) are associated with NVT use across urbanicity levels (all p < 0.05).

**Conclusions:**

This work suggests that active transport behaviors of rural and urban living adolescents are influenced by different neighborhood environment characteristics. Therefore, programs targeting active transport should consider specifics of the rural or urban context. Further, there is little overlap between factors associated with walking and NVT use, highlighting different needs based on transport mode choice.

